# Correlation of Blood Leukocytes, CRP, LDH, and Cytokine Levels with Disease Severity in Children with Adenovirus Pneumonia

**DOI:** 10.3390/v18030364

**Published:** 2026-03-16

**Authors:** Yuqun Wei, Xia Liu, Guangwan Lian, Ning Han, Yi Chen, Yingying Zhang, Wanli Liang, Xiaotong Zhan, Bing Zhu, Mingqi Zhao

**Affiliations:** 1 Center Laboratory, Guangzhou Women and Children’s Medical Center, Guangzhou Medical University, No 318 Renminzhong Road, Yuexiu District, Guangzhou 510120, China; wyq@stu.gzhmu.edu.cn (Y.W.); 15089163405@163.com (X.L.); guangwan78@163.com (G.L.);; 2 Army Medical Center of PLA, Daping Hospital, Chongqing 400042, China

**Keywords:** adenovirus pneumonia, pediatric patient, cytokines, disease severity

## Abstract

Objective: This study aims to investigate the correlation between blood leukocyte, CRP, LDH, and cytokine levels and the severity of illness in children with adenovirus pneumonia. Methods: A total of 100 children with adenovirus pneumonia (55 mild cases and 45 severe cases) who were treated at Guangzhou Women and Children’s Medical Center from January 2022 to January 2024, and 40 healthy children as a control group, were selected. Clinical data, some laboratory test data, and serum cytokine levels detected by flow cytometry were collected, and statistical methods were used to analyze the correlation between relevant indicators and the severity of the illness. Results: The research showed that among general clinical manifestations, the proportions of children with fever, dyspnea, pleural effusion, and moist rales in the severe group were all higher than those in the mild group (*p* < 0.05). Among the collected laboratory test data, indicators such as WBC, neutrophils, and LDH were significantly higher than in the mild group and the control group (*p* < 0.05) and were positively correlated with the severity of the disease. Regarding the tested cytokines, most children with adenovirus pneumonia showed elevated levels, and cytokines such as IL-6, IL-2, and IL-8 were significantly positively correlated with the disease. In the ROC curve analysis, NEU 6.03 × 10^9^/L (sensitivity 82.2%, specificity 72.7%, AUC 0.830) and IL-6 41.823 pg/mL (sensitivity 75.6%, specificity 81.8%, AUC 0.833) demonstrated certain value in the early identification of children with severe disease. Conclusion: In this study, laboratory indicators (C-reactive protein, lactate dehydrogenase, neutrophils, etc.) and changes in the levels of specific cytokines (TNF-β, IL-2, IL-6, IL-8, etc.) in children with adenovirus pneumonia were closely related to the severity of the disease. Notably, neutrophil count and interleukin-6 were significantly positively correlated with disease severity and had high AUC values, suggesting they may be important parameters for early prediction of the progression of mild adenovirus infection to severe disease.

## 1. Introduction

Human adenoviruses (HAdV) are highly contagious pathogens that can cause a variety of diseases, such as acute respiratory disease (ARD), including upper respiratory tract infections, bronchitis, and pneumonia [1,2]. While most infections are mild and self-limiting, severe and even fatal cases in children and immunocompromised individuals are increasingly being reported [3,4]. Among children with community-acquired pneumonia, adenovirus exhibits particularly significant pathogenicity compared to other viral pathogens [5]. Given the potentially severe clinical consequences of HAdV infection, coupled with the lack of sufficiently specific clinical manifestations, early-stage HAdV infection may be confused with numerous other respiratory infections. Furthermore, the disease progresses rapidly, making the transition point from mild to severe illness difficult to predict. Early and accurate identification of high-risk patients with a propensity for severe disease, followed by timely intervention, holds critical clinical significance for improving pediatric prognosis and reducing mortality rates. Currently, the clinical diagnosis of HAdV infection primarily relies on viral nucleic acid detection (PCR) or viral antigen detection [6]; however, these methods primarily focus on the presence or absence of pathogens, offering limited guidance for assessing the host’s immune status and predicting disease severity. Therefore, a comprehensive systemic assessment of patients with HAdV infection, involving the integrated analysis of multidimensional information including clinical manifestations, laboratory investigations, and imaging characteristics, is of paramount importance. In recent years, advances in immunology and molecular biology techniques have demonstrated that dynamic alterations in multiple soluble factors within peripheral blood—including inflammatory cytokines, chemokines, acute phase proteins, and immune cell subsets—assist in assessing the severity of viral infectious diseases and the host’s immune response status. The inflammatory mediator profiles and immune cell compositions, shaped by variations in viral load, host genetic background, and immune status across different patients, exhibit marked differences according to disease severity. This gives rise to distinct patterns of “cytokine storms” or immunosuppressive states [7,8,9]. However, there remains a lack of targeted research data to substantiate the relationship between changes in blood cell counts, blood biochemical indicators, and cytokine levels in pediatric patients with adenovirus-induced pneumonia and the severity of their condition. Furthermore, there is insufficient evidence to determine whether these parameters possess potential value for early diagnosis or as indicators for predicting severe disease progression.

This study retrospectively collected general data, partial laboratory examination results, imaging features, and other related indicators of the included samples and prospectively studied the serum levels of five types of cytokines in the study sample, aiming to explore the changes in blood cell counts, blood biochemical indicators, and cytokine levels in pneumonia caused by adenovirus infection in children, and to analyze the potential diagnostic value of these parameters.

## 2. Materials and Methods

### 2.1. Materials

#### 2.1.1. General Information

The study sample comprised pediatric patients presenting with community-acquired pneumonia at Guangzhou Women and Children’s Medical Center from January 2022 to January 2024. Inclusion criteria: (1) meeting pneumonia diagnostic criteria; (2) confirmed adenovirus infection via viral nucleic acid polymerase chain reaction (PCR) or gene sequencing of bronchoalveolar lavage fluid or blood samples, with no co-infections; and (3) age ≤ 15 years. Exclusion criteria: presence of other infectious diseases, chronic lung disease, chronic kidney disease, immunodeficiency disorders, congenital heart disease, hematologic disorders, recent surgery or trauma, current malignancy, or psychiatric health issues. A total of 100 pediatric patients with adenovirus pneumonia were enrolled. Specimens were categorized into severe and mild groups based on disease severity. Severe cases were classified according to the Technical Guidelines for Prevention and Control of Human Adenovirus Respiratory Infections (2019 Edition). Patients exhibiting typical clinical manifestations of human adenovirus pneumonia and meeting any of the following criteria were diagnosed with severe adenovirus pneumonia:

(1) Persistent high fever exceeding 39 °C for 5 consecutive days or more, accompanied by severe and frequent coughing;
(2) Heart rate exceeding 100 beats per minute, or respiratory rate reaching 30 breaths per minute or higher;
(3) Rapid progression of pulmonary lesions on imaging studies, with consolidation involving multiple lobes or segments;
(4) Arterial oxygen partial pressure (PaO_2_) below 70 mmHg, or pulse oxygen saturation (SpO_2_) below 90%, with no improvement in PaO_2_ levels despite supplemental oxygen or mask oxygen therapy;
(5) Condition severe enough to require endotracheal intubation and mechanical ventilation support;
(6) Presence of septic shock requiring vasopressors to maintain vital signs despite aggressive fluid resuscitation.

A total of 55 children with mild symptoms and 45 with severe symptoms were enrolled. Additionally, 40 healthy children who had not experienced respiratory infections, fever, or other illnesses within the preceding month were selected as controls.

As this study recruited participants aged 15 years or younger (minors), all legal guardians provided written informed consent. Prior to signing the consent form, guardians fully understood the study’s purpose, procedures, potential risks, and data usage, and voluntarily agreed to their child’s participation.

The research protocol received approval from the Ethics Committee of the Guangzhou Women and Children’s Medical Center (Approval No. Sui Fu Er Ke Lun Pi Zi [2022] No. 159A01).

#### 2.1.2. Data Collection

Data on the study subjects were collected from the electronic medical record (EMR) system, including patient demographics, clinical symptoms, selected laboratory findings, radiological findings, key therapeutic interventions, and final outcomes. Laboratory test data and imaging characteristics of the study subjects were collected from relevant tests performed on the first day of hospitalization.

#### 2.1.3. Cytokine Selection and Detection of Serum Cytokine Levels

Based on current research into pediatric adenovirus pneumonia, the selection of the 14 cytokines for this experiment was primarily guided by their distinct immunological roles. Pro-inflammatory cytokines, such as IL-1β, TNF-α, TNF-β, IL-6, and IL-8, are key drivers of the early inflammatory response [7]. Th1-type cytokines, including IFN-γ, IL-2, and IL-12p70, are responsible for mediating cellular antiviral immunity [8]. Th2-type cytokines, such as IL-4 and IL-5, are associated with humoral immunity and allergic reactions [9]. Regulatory cytokines, for instance, IL-10, are crucial for maintaining immune homeostasis [10]. Finally, Th17-type cytokines, including IL-17A, IL-17F, and IL-22, are linked to neutrophil infiltration and tissue damage [11,12]. This comprehensive panel of cytokines facilitates a systematic analysis of the dynamic evolution of the immune microenvironment in pediatric adenovirus pneumonia, which is of significant importance for clarifying the disease’s progression and identifying potential therapeutic targets. The kit used in this study (purchased from Tianjin Kuangbotongsheng Biotechnology Co., Ltd., Tianjin, China, batch number 20231016) is based on the principle of immunofluorescence luminescence technology. It utilizes distinct microsphere populations, each labeled with varying fluorescent intensities, and coats their surfaces with specific antibodies corresponding to cytokines such as IFN-γ, IL-1β, IL-2, IL-4, IL-5, IL-6, IL-8, IL-10, IL-12p70, IL-17A, IL-17F, IL-22, TNF-α, and TNF-β to serve as capture microspheres. After these microspheres bind to their respective cytokines in the sample, a biotin-labeled detection antibody is added, forming an immune complex structure composed of an antibody-capture microsphere-target cytokine–biotin-labeled detection antibody. Subsequently, PE (phycoerythrin)-labeled streptavidin (SA-PE) is added to the system. This avidin specifically binds to biotin, enabling the entire complex to be detected in the PE fluorescence channel of a flow cytometer. Based on the differing fluorescence intensities (PE fluorescence) exhibited by each immune complex, a quantitative analysis of the specific concentration of each cytokine in the sample can be performed by referencing a standard curve.

All study subjects, including children in both the case and control groups, had 2 mL of venous blood collected on the morning of their first day of hospitalization under fasting conditions. The samples were placed into dry tubes. After being allowed to stand at room temperature for 60 min, the samples were centrifuged at 1500 revolutions per minute for 10 min. Subsequently, the separated serum was transferred to sterile EP tubes and stored in a −80 °C freezer for future use. During the experiment, we utilized a flow cytometer to precisely measure the levels of 14 cytokines, including IL-2, IL-4, IL-6, IL-10, TNF, IFN-γ, and IL-17A, among others, in the serum. The entire operational procedure was conducted in strict accordance with the standard operating procedures provided by the Kuangbo company for the cytokine detection kit.

With the BD FACS Canto II flow cytometer properly calibrated, we employed BD FACSDiva software(Version 8.0.3) to gather data from each sample. Subsequently, FCAP Array v3 software was used to analyze the results, generating standard curves for each cytokine level and, from there, determining the concentration of target cytokines present in each sample.

#### 2.1.4. Statistical Analysis

The SPSS 26.0 software was used for the statistical analysis of the data. Count-type data are presented as the number of cases and their percentages (%), and the chi-squared test (χ^2^ test) was used to assess the differences between groups. The experimental data were first verified for normal distribution characteristics using the Shapiro–Wilk test method. For continuous measurement data that conformed to a normal distribution, we describe them using the mean ± standard deviation; for measurement data that did not conform to a normal distribution, the median and quartiles were selected to represent them. In cases involving multiple comparisons, where the study data conformed to a normal distribution and the overall variances of all sample groups were homogeneous, one-way analysis of variance (ANOVA) was employed, followed by post hoc comparisons (using LSD for homogeneous variances and Tamhane’s method for heterogeneous variances). Where data did not conform to a normal distribution, the Kruskal–Wallis H test was used, with post hoc comparisons conducted via Dunn’s test. Meanwhile, Spearman’s rank correlation analysis was utilized to investigate the strength of the association between various indicators and the severity of the condition. Additionally, receiver operating characteristic (ROC) curves were constructed to evaluate the diagnostic efficacy of different indicators. Finally, a *p*-value < 0.05 was set as the standard threshold for determining that the results had statistically significant differences.

## 3. Results

### 3.1. Clinical Characterization of the Three Groups of Children

According to the inclusion criteria of this study, we enrolled 100 confirmed pediatric patients and selected 40 healthy children from the same period. General characteristics and clinical data are presented in Table 1. Males constituted a larger proportion in the study: specifically, 39 males (71%) in the mild group, 28 males (62%) in the severe group, and 29 males (73%) in the control group. No significant differences were observed between the three groups in terms of age or gender (*p* > 0.05). Regarding clinical manifestations, the children presented with symptoms including fever, cough, rhinorrhea, and expectoration, with fever and cough being the most common. The proportion of children in the severe group exhibiting fever, dyspnea, pleural effusion, and moist rales was higher than that in the mild group (*p* < 0.05).

### 3.2. Analysis of Routine Laboratory Test Indicators in the Three Groups of Children

During inpatient management, laboratory tests are frequently employed to assess a child’s condition. In this study, selected hematological parameters (white blood cell count, absolute lymphocyte count, C-reactive protein, etc.), biochemical parameters (ALT, AST, LDH, albumin, etc.), and blood gas analysis parameters (pH, PaO_2_, and PaCO_2_) were collected. These parameters provide preliminary insights into the child’s inflammatory response, liver function, cardiac function, and acid–base balance, thereby informing clinical decision-making and evaluation.

Among the routine laboratory test indicators of the three groups of children, the white blood cell count, absolute values of neutrophils and monocytes, the neutrophil and lymphocyte ratios, and the levels of AST, LDH, γ-GT, globulin, PH, PCO_2_, albumin, and creatinine showed differences (*p* < 0.05) among the healthy control group, the mild group, and the severe group, as determined by one-way ANOVA. The PCT and CRP levels in the severe group were significantly higher than those in the mild group (*p* < 0.05) (see Table 2). Post hoc comparisons were performed on the indicators with significant differences; the severe group exhibited significantly higher levels of WBC, neutrophil, MONO#, neutrophil%, and LDH compared to both the mild group and the control group (all *p* < 0.05), as shown in Table 3.

### 3.3. Detection of Serum Cytokines in the Three Groups of Children

**Figure 1:**
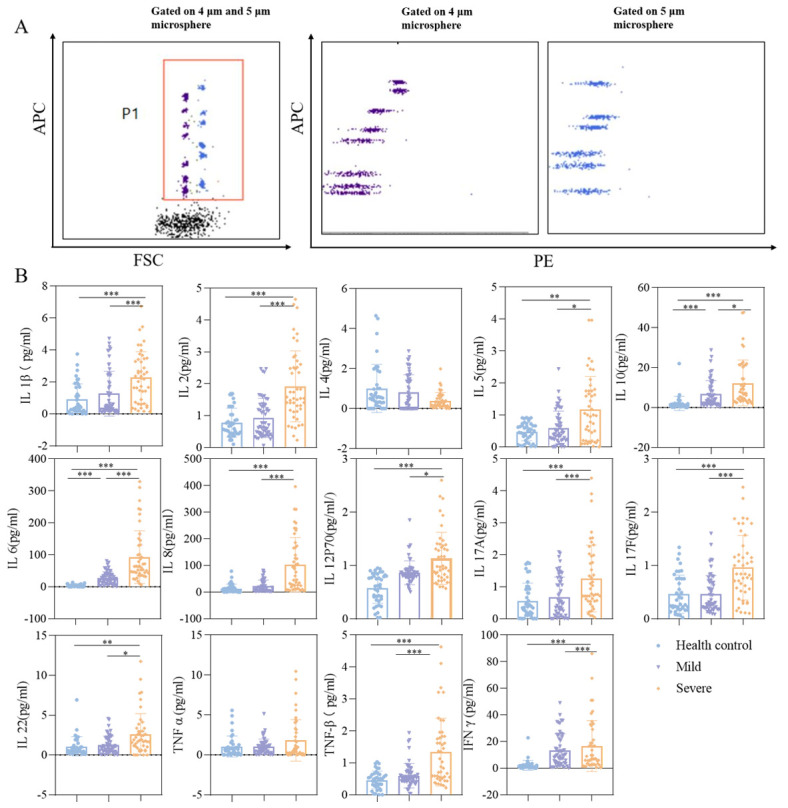
Serum cytokine assay in children with adenovirus pneumonia: (**A**) FACS dot plot showing that microspheres were classified according to forward scattered light (FSC), including two groups of size, and different cytokines were classified according to APC fluorescence intensity, and the strength of PE fluorescence intensity represented the level of cytokines. (**B**) Box plots displaying the distribution of serum cytokine levels (IFN-γ, IL-1β, IL-6, IL-8, IL-10, IL-12p70, IL-17F, IL-22, TNF-α, and TNF-β) in children with adenovirus pneumonia. Individual data points are overlaid, with colors indicating groupings: blue dots for healthy controls (*n* = 40), purple triangles for mild cases (*n* = 55), and orange dots for severe cases (*n* = 45). Data analysis—intergroup comparisons were conducted using the Kruskal–Wallis H test. Where the overall test yielded statistically significant results (*p* < 0.05), Dunn’s post hoc multiple comparisons were performed, with Bonferroni correction applied to control for Type I errors. Comparison of serum levels of IFN-γ, IL-1β, IL-6, IL-8, IL-10, IL-12p70, IL-17F, IL-22, TNF-α, and TNF-β levels were compared. *p* < 0.05 (*), *p* < 0.01 (**), *p* < 0.001 (***) indicate statistically significant differences.

Multiple soluble factors in peripheral blood, such as inflammatory cytokines, chemokines, and acute-phase proteins, have been demonstrated to aid in assessing the severity of viral infectious diseases and the host’s immune response status. Adenovirus infection is also associated with various inflammatory cytokines. We measured the levels of certain cytokines. The cytokine data for the three cell groups are presented in Table 4. Figure 1 displays the results of serum cytokine detection in pediatric patients with adenovirus pneumonia. (A) The FACS dot plot illustrates the classification of microspheres based on forward scatter light (FSC), revealing two distinct populations of different sizes. Different cytokines are categorized according to their APC fluorescence intensity, while the intensity of PE fluorescence indicates the presence of cells. (B) A comparative analysis of IFN-γ, IL-1β, IL-6, IL-8, IL-10, IL-12p70, IL-17F, IL-22, TNF-α, and TNF-β levels in the serum of children across the different experimental groups is provided.

In this study, following adenovirus infection, IL-1β, IL-2, IL-6, IL-8, IL-12P70, IL-17A, IL-17F, IFN-γ, and TNF-β all exhibited varying degrees of elevation. This was particularly pronounced in the severe pneumonia group, where the elevation of various pro-inflammatory cytokines showed statistical differences compared to the mild group and the control group (*p* < 0.05).

### 3.4. Imaging Features

High-resolution chest CT scans can clearly demonstrate the location, extent, morphology, and severity of pulmonary lesions in patients with pneumonia. All 100 enrolled pediatric patients underwent chest CT on the first day of hospitalization. Among them, bronchopneumonia imaging features were most prevalent in the moderate-to-severe group (54.5% vs. 42.2%). Pulmonary consolidation was significantly more common in the severe group (33.4%) than in the mild group (1.8%), with a statistically significant difference (*p* < 0.001); see Table 5.

### 3.5. Therapy

The primary nebulization therapy for mild cases was method 1 (40%); for severe cases, it was method 2 (100%). The proportions of severe cases receiving methylprednisolone, fiberoptic bronchoscopy, and gamma globulin therapy were all higher than those for mild cases (*p*< 0.05) (Table 6).

### 3.6. Outcome

Following treatment, 54 (98.2%) children in the mild group and 37 (82.2%) in the severe group improved and were discharged. One (1.8%) child in the mild group and eight (17.8%) in the severe group were transferred to intensive care (*p* = 0.006). See Table 7.

### 3.7. Correlation Analysis of WBC, CRP, LDH, and Cytokine Levels with Disease Extent

**Figure 2:**
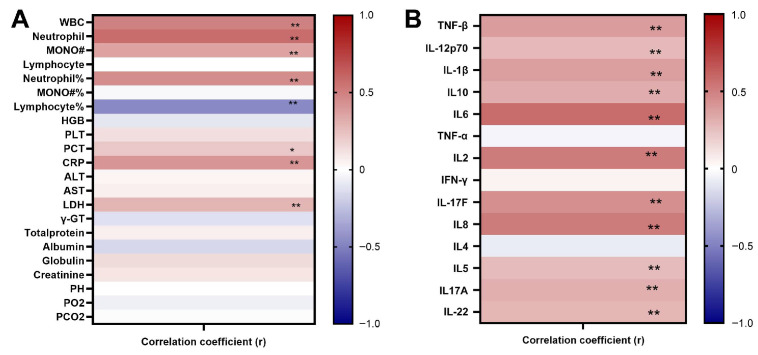
Correlation analysis between disease severity and clinical laboratory indicators and cytokines. The heatmap in (**A**) displays Spearman correlation coefficients between clinical laboratory indicators and disease severity. The heatmap in (**B**) displays Spearman correlation coefficients between cytokines and disease severity. Red indicates a positive correlation (r > 0), blue denotes a negative correlation (r < 0), and white signifies no correlation (r = 0). Color bar values range from −1.0 to 1.0. * *p* < 0.05, ** *p* < 0.01.

Spearman’s correlation analysis revealed that multiple laboratory parameters and cytokine concentrations in pediatric adenovirus cases were closely associated with disease severity progression. Specifically, white blood cell count (WBC), absolute neutrophil count and percentage, lactate dehydrogenase (LDH), and C-reactive protein (CRP) levels demonstrated significant positive correlations with disease severity, while levels of IL-6, IL-2, IL-8, IL-17F, and TNF-β also demonstrated significant positive correlations with disease severity. Notably, both the absolute neutrophil count and IL-6 levels exhibited strong correlations with disease severity. See Figure 2.

### 3.8. ROC Curve Analysis of Multi-Indicator Joint Detection

**Figure 3:**
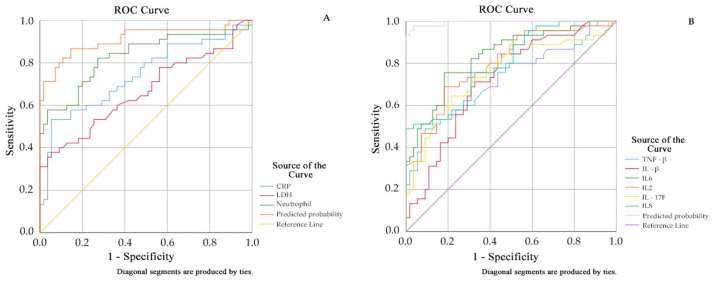
The figure on the left (**A**) shows the role of routine blood and blood biochemistry levels in predicting the progression of adenoviral infection from mild to severe disease; the figure on the right (**B**) shows the role of cytokine levels in predicting the progression of adenoviral infection from mild to severe disease.

Multivariate logistic regression analysis: Parameters with statistical significance (*p* < 0.05) from Table 3 (P_23_) and Figure 1B were employed as independent variables, with disease severity grouping serving as the dependent variable. Variable selection was carried out using stepwise regression, setting the entry criterion at α = 0.05 and the exclusion criterion at α = 0.1. ROC curves were plotted for statistically significant indicators identified in the logistic regression analysis. See Table 8, Table 9 and Figure 3.

## 4. Discussion

ADV is one of the severe forms of community-acquired pneumonia in children and poses a significant threat to their health [4]. This study evaluated multiple disease indicators in pediatric patients with adenovirus pneumonia, including general clinical characteristics, blood biochemistry, imaging features, and cytokines. It comprehensively analyzed differences in various indicators across different groups. General clinical features: Multiple studies have confirmed that children with severe adenoviral pneumonia frequently present with persistent high fever and markedly worsening respiratory distress [13]. In this study, most severe cases also exhibited clinical manifestations such as pleural effusion and moist rales. This may be attributed to an excessive inflammatory response leading to diffuse pulmonary inflammatory infiltration and increased alveolar-capillary permeability.

In recent years, blood cell counts and biochemical indicators in blood have been widely applied in the diagnosis of infectious diseases, demonstrating high diagnostic value [6]. The findings of this study indicate that white blood cell count is significantly positively correlated with disease severity. The findings of Lin et al. [13] show that patients infected with adenovirus type 2 are more likely to exhibit elevated white blood cell counts (>15 × 10^9^/L), whereas those infected with adenovirus type 7 more frequently present with reduced white blood cell counts (<5 × 10^9^/L). This suggests that different adenovirus serotypes may influence the host immune response through distinct mechanisms. Neutrophils, as a key component of the human immune system, phagocytose and kill pathogens, representing the second line of defense for the host against invading pathogens and providing a powerful protective mechanism for the body [14]. In most instances, neutrophils mount a rapid response upon encountering microbial pathogens like bacteria, fungi, and parasites [15]. In this study, the absolute neutrophil count and percentage showed a significant positive correlation with disease severity, alongside a high AUC value and sensitivity in the ROC curve, suggesting their potential as a sensitive screening tool for severe adenovirus infection. Neutrophilia is a common manifestation of systemic inflammation during viral infections. The elevated neutrophil counts observed in severe cases may be associated with an excessive inflammatory response induced by adenovirus replication, consistent with findings from previous studies on viral respiratory infections [16].

In this study, levels of lactate dehydrogenase (LDH), C-reactive protein (CRP), and procalcitonin (PCT) showed a significant positive correlation with disease severity. The association between these three indicators and disease severity has been confirmed by multiple studies [17,18,19]. LDH is widely distributed in tissues such as myocardium, liver, lungs, red blood cells, and skeletal muscle. Across various diseases, LDH serves as a crucial indicator reflecting disease severity and prognosis. Wang et al.’s study [20] demonstrated that serum LDH concentrations in children with respiratory infections were significantly higher than in healthy controls, with pneumonia patients exhibiting higher levels than those with upper respiratory tract infections. C-reactive protein (CRP) is produced by the liver in response to inflammatory cytokines such as interleukin-6 (IL-6). It is an acute-phase reactant protein that generally rises rapidly within a few hours after tissue injury, the onset of infection, or other inflammatory causes [21]. Shen et al. [22] believe that the increase in CRP levels in children infected with adenovirus indicates that adenovirus infection can trigger an inflammatory response similar to that caused by bacterial infection. The increase in CRP levels may show serotype-specific characteristics, which is reflected in the fact that children infected with adenovirus type 3 often show higher CRP values compared to children with other adenovirus serotypes [23]. Procalcitonin (PCT) is a hormone secreted by thyroid cells, and its level changes are closely related to the severity of infection [1]. Regarding radiographic features, Wang et al. [20] found that bilateral multilobar pulmonary consolidation with prominent bronchial air signs serves as an independent predictor of severe ADVP. In this study, the proportion of children with pulmonary consolidation in the severe group was significantly higher than in the mild group, potentially aiding in the early identification of severe cases.

Cytokines play a pivotal role in the pathogenesis of adenovirus infection, and the findings of this study highlight the diagnostic value of several key cytokines. Th1 cells, CD4+ T cells, macrophages, and dendritic cells are all cell types capable of secreting pro-inflammatory cytokines [24]. When the body is infected by a virus, the immune system responds rapidly, one manifestation being an elevation in pro-inflammatory cytokine levels [25]. In this study, pediatric patients exhibited a significant positive correlation between pro-inflammatory factor levels (such as IL-1β, TNF-β, IL-6, and IL-8) and disease severity, consistent with the mechanism of immune system activity during viral infection. When the body is subjected to a viral infection, the immune system responds rapidly, one of which is the elevation of pro-inflammatory cytokines. The increase in these cytokines is a defense mechanism of the body against viral invasion, aiming to activate the immune system and clear the virus. However, an excessive inflammatory response can also lead to tissue damage and the development of disease. This finding reveals the complex immune response triggered by adenovirus infection and emphasizes the key role of these cytokines in the course of the disease.

Increasing evidence suggests that more severe disease is associated with the tissue accumulation of macrophages and the production of inflammatory cytokines such as IL-1β, IL-6, and TNF-α [26]. The findings of this study indicate that serum IFN-γ levels in critically ill patients were significantly elevated compared to both the mildly ill group and healthy controls. IFN-γ is the most typical Th1-type cytokine, has a significant pro-inflammatory effect and antiviral activity, and during viral infection, it enables the body to respond more quickly to viral infection by promoting the occurrence of inflammatory responses [27,28]. The inflammatory response can recruit more immune cells to the site of infection and release various inflammatory mediators to jointly defend against viral invasion. This study found that IL-17A, IL-6, IL-17F, and IL-22 were significantly positively correlated with disease severity. Among them, Th17 cells are a group of CD4+ effector T cells recently identified in research, which can secrete a series of cytokines, including IL-17A, IL-17F, IL-6, and IL-22 [29], the most important of which is IL-17A, which has a strong pro-inflammatory effect and can participate in the complex regulatory process of the body’s immune defense system together with Th1, Th2, and regulatory T cells [30]. Studies have found that IL-17A can enhance TNF-induced IL-6 expression, thereby activating the immune response [29]. Ma et al. [31] also found that IL-17 can promote astrocytes to express IL-6, producing a synergistic effect with IL-6. However, the findings of this study reveal that the decrease in IL-4 may lead to a reduction in the body’s ability to clear the virus. As IL-4 is an important Th2-type cytokine, it primarily participates in regulating immune responses, promoting the proliferation and differentiation of B cells, and the production of IgE [32]. However, during the acute phase of adenovirus infection, due to the direct stimulation of the immune system by the virus, the body often mounts a strong Th1-type immune response to clear the virus. In this situation, the Th2-type immune response is suppressed to a certain extent, leading to a reduction in IL-4 production, which in turn causes its serum concentration to decrease. This reduction may further affect the activation of B cells and the production of antibodies, thus having a certain impact on the body’s antiviral capability.

During the acute phase of adenovirus infection, the levels of TNF-β and IL-6 in the serum of patients showed a significant increase. This rise may be attributed to the stimulation of immune-active cells, which triggers the massive secretion of TNF, which in turn induces the production of IL-6, thereby exacerbating the inflammatory response [33]. It has been reported that in children with severe pneumonia, macrophages synthesize large amounts of TNF (tumor necrosis factor), and by secreting this cytokine, they cause direct damage to vascular endothelial cells. This damage aggravates the extent of structural and functional destruction of lung tissue. At the same time, the damaged lung tissue will further trigger the release of more TNF factors, forming a mutually reinforcing vicious cycle. This cycle continuously intensifies, leading to recurrent pneumonia that is difficult to completely cure [34]. Furthermore, this study found that IL-10 exhibited a significant positive correlation with disease severity, with elevated IL-10 concentrations observed in comparison to both the control group and children with mild disease. IL-10 is an anti-inflammatory cytokine [35] whose functions include the regulation of the immune response and the suppression of inflammation. During the acute phase of adenovirus infection, in order to counter the viral invasion, the immune system releases a large number of pro-inflammatory cytokines, such as TNF-β and IL-6. To balance this intense inflammatory response, the body simultaneously produces more IL-10.

Comparison with Other Viral Pneumonias: In patients with severe pneumonia induced by influenza A viruses, levels of IL-6, IFN-γ, and IL-2 are markedly elevated [36]. Furthermore, in pediatric cases of respiratory syncytial virus (RSV) pneumonia, IL-13, IL-6, IL-8, IL-10, and TNF-α have been identified as independent predictors for assessing disease severity [37].

In summary, in practical application, a comprehensive assessment incorporating multiple indicators is required to achieve the early identification of critically ill pediatric patients.

This study has several limitations. First, adenovirus serotype is associated with disease severity; however, the specific serotype was not identified in this study. Second, as a single-center investigation with a limited sample size, the findings necessitate validation through multi-center studies with larger cohorts. Although the five cytokines measured span five distinct functional classes, the overall number of analytes was restricted. Furthermore, the cross-sectional design of this study allows for only a snapshot of cytokine levels at a single time point, precluding an analysis of their dynamic evolution and causal relationships throughout the disease course. Future prospective cohort studies could address this. Additionally, integrating cutting-edge omics technologies, such as metagenomics and single-cell sequencing, could enable a system-level analysis of host–pathogen interactions. This approach holds promise for elucidating the pathogenic mechanisms of adenovirus infections and identifying novel therapeutic targets.

## 5. Conclusions

In this study of pediatric adenovirus pneumonia patients, laboratory indicators (C-reactive protein, lactate dehydrogenase, neutrophils, etc.) and specific cytokine levels (TNF-β, IL-2, IL-6, IL-8, etc.) were closely correlated with disease severity. Notably, neutrophil counts and interleukin-6 exhibited a significant positive correlation with disease severity and demonstrated higher AUC values, suggesting they may serve as important parameters for early prediction of progression from mild to severe adenovirus infection.

## Figures and Tables

**Table 1:** Demographic and clinical characteristics of the three groups of children.

Characteristic	Mild (*n* = 55)	Severe (*n* = 45)	Health Control (*n* = 40)	*p*
Age (months)	48.53 ± 30.33	42.38 ± 29.41	48 ± 25.32	>0.05
Sex, (male,%)	39 (71%)	28 (62%)	29 (73%)	>0.05
Age group				
<=0.5 y	1 (1.8%)	0		>0.05
0.5–3 y	18 (32.7%)	21 (46.7%)	--	
3–6 y	26 (47.3%)	14 (31.1%)	--	
>6 y	10 (18.2%)	10 (22.2%)	--	
Symptoms				
Fever	41 (74.5%)	45 (100%)	--	<0.001
Cough	45 (84.8%)	40 (88.9%)	--	>0.05
Rhinorrhea	35 (67.3%)	35 (77.8%)	--	>0.05
Expectoration	38 (69.1%)	38 (84.4%)	--	>0.05
Nasal congestion	30 (54.5%)	29 (64.4%)	--	>0.05
Dyspnea	0 (0%)	34 (75.5%)	--	<0.001
Pleural effusion	0 (0%)	6 (13.3%)	--	0.005
Moist rale	21 (38.2%)	45 (100%)	--	<0.001
Tonsillitis	36 (65.5%)	31 (68.9%)	--	>0.05

Note: Data analysis—Continuous variables with normal distribution are expressed as mean ± standard deviation. Comparisons between three groups were conducted using one-way analysis of variance. Count data are presented as case numbers and percentages (%), with differences between groups assessed using the chi-squared test (χ^2^ test). No adjustment was made for multiple testing.

**Table 2:** Laboratory indicators for children in the viral pneumonia group and the control group.

Characteristic	Health Control (*n* = 40)	Mild (*n* = 55)	Severe (*n* = 45)	F/H	*p*
Blood routine					
WBC (10^9^/L)	6.77 ± 1.69	9.48 ± 3.99	15.50 ± 6.59	41.29	<0.01
Neutrophil (10^9^/L)	3.08 ± 1.21	4.94 ± 2.31	10.62 ± 5.16	61.08	<0.01
MONO# (10^9^/L)	0.44 ± 0.15	0.93 ± 0.40	1.53 ± 0.87	41.12	<0.01
Lymphocyte (10^9^/L)	1.08 ± 0.17	2.76 ± 0.37	3.26 ± 1.88	0.75	0.382
Neutrophil%	44.80 ± 12.46	52.85 ± 14.99	65.96 ± 13.49	25.65	<0.01
MONO#%	7.60 ± 6.90	9.80 ± 3.17	9.42 ± 3.39	2.86	0.061
Lymphocyte%	43.35 ± 12.94	34.93 ± 14.44	22.58 ± 11.61	27.01	<0.01
HGB (g/L)	120.68 ± 29.28	114.13 ± 12.93	110.76 ± 12.51	2.97	0.05
PLT (×10^9^/L)	293.93 ± 86.88	313.11 ± 111.35	351.20 ± 136.03	2.84	0.07
Blood biochemistry					
ALT (U/L)	12 (12, 16)	14 (10, 17)	14 (10, 18)	1.55	0.93
AST (U/L)	29 (27, 33.25)	34 (29, 37)	33 (29, 40)	1.70	<0.01
LDH (U/L)	244 (230.50, 249.75)	270 (234, 293)	293 (262, 410.50)	8.98	<0.01
γ-GT (U/L)	10 (8, 13)	12 (10, 15)	11 (9, 14)	1.84	<0.01
Total protein (g/L)	68.81 ± 4.11	66.76 ± 5.00	66.76 ± 6.43	2.15	0.12
Albumin (g/L)	43.34 ± 2.57	40.21 ± 2.91	38.80 ± 4.45	19.59	<0.01
Globulin (g/L)	25.47 ± 2.74	26.45 ± 4.29	27.96 ± 5.25	3.72	0.03
Creatinine (mg/dL)	36.23 ± 9.29	26.62 ± 8.81	28.18 ± 8.21	15.13	<0.01
PCT (ng/mL)	--	0.17 (0.10, 0.77)	0.3 (0.1, 1.37)	--	0.30
CRP (mg/L)	--	20.16 (12.3, 39.15)	63.98 (23.33, 92.48)	--	<0.01
Blood gas analysis					
PH	7.34 ± 0.03	7.41 ± 0.48	7.40 ± 0.05	28.42	<0.01
PO_2_	8.72 ± 4.88	8.89 ± 3.93	8.46 ± 3.93	0.11	0.9
PCO_2_	5.00 ± 1.17	5.04 ± 1.31	6.17 ± 0.94	14.32	<0.01

Note: Data analysis—normally distributed data are expressed as mean ± standard deviation; non-normally distributed data are presented as median (interquartile range). For data meeting normality and homogeneity of variances across sample groups, one-way ANOVA was employed; for non-normally distributed data, the Kruskal–Wallis H test was used. No adjustment was made for multiple testing. F/H denotes the ratio of between-group variation attributable to the treatment factor to within-group variation attributable to random error. P represents the probability of observing the current sample statistic (F-value or H-value) or a more extreme result under the assumption that the null hypothesis (H_0_) holds. Abbreviations: WBC, white blood cell; MONO, monocyte count; HGB, hemoglobin; PLT, platelet; ALT, alanine aminotransferase; AST, aspartate aminotransferase; LDH, lactate dehydrogenase; γ-GT, γ-glutamyl transferase; PCT, procalcitonin; CRP, C-reactive protein; PH, potential of hydrogen; PO_2_, partial pressure of oxygen; PCO_2_, partial pressure of carbon dioxide.

**Table 3:** Post hoc comparisons.

Characteristic	*p*		
WBC	P_12_ = 0.005	P_13_ < 0.001	P_23_ < 0.001
Neutrophil	P_12_ = 0.008	P_13_ < 0.001	P_23_ < 0.001
MONO#	P_12_ < 0.001	P_13_ < 0.001	P_23_ < 0.001
Neutrophil%	P_12_ = 0.006	P_13_ < 0.001	P_23_ < 0.001
Lymphocyte%	P_12_ = 0.003	P_13_ < 0.001	P_23_ < 0.001
AST	P_12_ = 0.018	P_13_ = 0.426	P_23_ = 0.588
LDH	P_12_ < 0.001	P_13_ = 0.007	P_23_ = 0.033
γ-GT	P_12_ = 0.161	P_13_ = 0.152	P_23_ = 0.903
Albumin	P_12_ < 0.001	P_13_ < 0.001	P_23_ = 0.198
Globulin	P_12_ = 0.445	P_13_ = 0.020	P_23_ = 0.327
Creatinine	P_12_ < 0.001	P_13_ < 0.001	P_23_ = 0.378
PH	P_12_ < 0.001	P_13_ < 0.001	P_23_ = 0.985
PCO_2_	P_12_ < 0.001	P_13_ < 0.001	P_23_ = 0.896

Note: Data analysis—post hoc pairwise comparisons were conducted for statistically significant indicators (*p* < 0.05) in Table 2. When data were normally distributed with equal variances, LSD tests were applied; when variances were unequal, Tamhane’s test was used. For non-normally distributed data, Dunn’s test was employed. P_12_ represents the comparison between the control group and the mild group, P_13_ represents the comparison between the control group and the severe group, and P_23_ represents the comparison between the mild group and the severe group.

**Table 4:** Cytokine data for the three groups.

Characteristic	Health Control (*n* = 40)	Mild (*n* = 55)	Severe (*n* = 45)
TNF-β	0.49 (0.28, 0.64)	0.49 (0.41, 0.68)	0.95 (0.53, 1.81)
IL-12p70	0.62 (0.34, 0.81)	0.81(0.77, 0.90)	0.96 (0.77, 1.37)
IL-1β	0.43 (0.16, 1.67)	0.52 (0.21, 2.10)	2.21 (0.66, 3.44)
IL-10	1.17 (0.92, 1.91)	3.61 (1.81, 10.32)	7.05 (3.67, 18.85)
IL-6	2.24 (0.76, 4.29)	22.89 (10.49, 37.73)	61.49 (42.43, 122.61)
TNF-α	0.76 (0.16, 1.19)	0.83 (0.33, 1.49)	0.49 (0.15, 2.66)
IL-2	0.68 (0.44, 1.03)	0.68 (0.42, 1.34)	1.54 (1.14, 2.52)
IFN-γ	1.19 (0.50, 2.14)	6.93 (2.69, 18.4)	9.82 (3.70, 23.66)
IL-17F	0.31 (0.19, 0.75)	0.28 (0.20, 0.68)	0.83 (0.50, 1.22)
IL-8	8.23 (2.53, 19.19)	11.91 (3.95, 41.13)	81.41 (20.47, 171.70)
IL-4	0.56 (0.20, 1.69)	0.42 (0.00, 1.52)	0.30 (0.15, 0.51)
IL-5	0.51 (0.22, 0.71)	0.43 (0.23, 0.86)	1.00 (0.20, 1.75)
IL-17A	0.37 (0.12, 0.93)	0.44 (0.11, 1.18)	0.77 (0.55, 1.99)
IL-22	0.72 (0.35, 1.72)	0.91 (0.45, 2.11)	1.82 (0.91, 2.99)

Note: Non-normally distributed data are presented as median (interquartile range). Abbreviations: TNF-β, tumor necrosis factor-beta; IL-12p70, interleukin-12p70; IL-1β, interleukin-1beta; IL-10, interleukin-10; IL-6, interleukin-6; TNF-α, tumor necrosis factor-alpha; IL-2, interleukin-2; IFN-γ, interferon-gamma; IL-17F, interleukin-17F; IL-8, interleukin-8; IL-4, interleukin-4; IL-5, interleukin-5; IL-17A, interleukin-17A; IL-22, interleukin-22.

**Table 5:** Comparison of imaging features between the severe group and the mild group.

	Mild (*n* = 55)	Severe (*n* = 45)	*p*
No abnormalities	4 (7.3%)	0 (0%)	0.07
Bronchitis	20 (36.4%)	11 (24.4%)	0.20
Bronchopneumonia	30 (54.5%)	19 (42.2%)	0.22
Pulmonary consolidation	1 (1.8%)	15 (33.4%)	<0.001

Note: ‘No abnormalities’ indicates no significant radiographic abnormalities. Bronchitis involves the bronchi and bronchioles, primarily manifesting as thickened bronchial walls and small nodules in the lobular centers. Bronchopneumonia involves bronchi and lobular alveoli, primarily manifesting as patchy consolidation along bronchial distribution and the tree-in-bud sign. Pulmonary consolidation involves segmental and lobar alveoli, primarily manifesting as extensive patchy consolidation and the air bronchogram sign. Data analysis—Count data are presented as the number of instances and their percentages (%), with the chi-squared test (χ^2^ test) employed to assess differences between groups.

**Table 6:** Comparison of therapy between the severe group and the mild group.

	Mild (*n* = 55)	Severe (*n* = 45)	*p*
Other symptomatic treatments	55 (100%)	45 (100%)	>0.05
nebulization therapy			
1	24 (43.6%)	0 (0%)	<0.001
2	22 (40%)	45 (100%)	<0.001
MPSS	1 (1.8%)	13 (28.9%)	<0.001
Bronchoscope	1 (1.8%)	7 (15.6%)	0.012
Gamma Globulin	2 (3.6%)	8 (17.8%)	0.019

Note: Other symptomatic treatments include antipyretics, cough suppressants, and expectorants; Nebulization therapy 1: salbutamol + ipratropium bromide + acetylcysteine; 2: salbutamol + ipratropium bromide + acetylcysteine + budesonide (only primary drugs listed); MPSS (methylprednisolone sodium succinate) is the corticosteroid therapy. Data analysis—count data are presented as the number of instances and their percentages (%), with the chi-squared test (χ^2^ test) employed to assess differences between groups.

**Table 7:** Comparison of the outcomes between the severe group and the mild group.

	Mild (*n* = 55)	Severe (*n* = 45)	*p*
Improvement	54 (98.2%)	37 (82.2%)	0.006
Intensive care	1 (1.8%)	8 (17.8%)	0.006

Note: Data analysis—count data are presented as the number of instances and their percentages (%), with the chi-squared test (χ^2^ test) employed to assess differences between groups.

**Table 8:** The value of laboratory detection indicators in differentiating between mild and severe groups of pediatric patients with adenovirus pneumonia.

	AUC	95%CI	Optimal Cutoff Point	Sensitivity	Specificity	Jordon Index
CRP	0.741	0.640–0.842	56.525	53.3%	94.5%	0.478
LDH	0.666	0.555–0.776	342.5	37.8%	94.5%	0.323
NEU	0.830	0.745–0.915	6.03	82.2%	72.7%	0.549
Unite	0.912	0.849–0.974	0.358	86.7%	85.5%	0.722

**Table 9:** The value of cytokines in differentiating between mild and severe adenovirus pneumonia in pediatric patients.

	AUC	95%CI	Optimal Cutoff Point	Sensitivity	Specificity	Jordon Index
TNF-β	0.724	0.622–0.826	0.984	48.9%	90.9%	0.398
IL-1β	0.721	0.621–0.820	0.524	84.4%	54.5%	0.389
IL-6	0.833	0.754–0.913	41.823	75.6%	81.8%	0.574
IL-2	0.799	0.711–0.886	1.387	68.9%	81.8%	0.507
IL-17F	0.754	0.655–0.852	0.709	64.4%	78.2%	0.426
IL-8	0.800	0.715–0.885	84.462	48.9%	100%	0.489
Unite	0.995	0.986–1.000	0.379	97.8%	96.4%	0.942

## Data Availability

All original contributions of this study are contained with in the main article. Requests for additional data clarification should be addressed to the corresponding authors.
